# The Editor's Letter-Box

**Published:** 1915-02-06

**Authors:** 


### THE EDITOR'S LETTER-BOX.

### The Hampstead Appointment.

*To the Editor of* The Hospital.

Sir,—Much indignation prevails among hospital officers at the action of the committee of the Hampstead General Hospital in appointing a secretary whose only qualification for the post would appear to be that he was once secretary to the Boy Scouts' Association. They feel it more acutely because the committee have not acted up to the letter of their official advertisement, which stated that candidates "must have had previous hospital experience."

All the candidates interviewed save only the one appointed were men of long experience in hospital affairs, and naturally they feel aggrieved that years of special training and hard work should count as naught, and they are wondering what effect it will have on junior officers—the would-be secretaries of to-morrow.

Already opinions have been freely expressed, and as one junior put it at a gathering of juniors for a lecture on hospital administration, "What is the use of giving the hospitals the best years of our lives if this is to be the reward meted out by hospital committees? Better that we strike out into something else while there is yet time." And on the face of it who can blame them for thinking thus?

Surely a committee has a duty to the public, and it would not appear that it was carrying it out properly in appointing a man who has to learn his business at the expense of the hospital, especially when there are several trained men to choose from.

Hospital officers feel that now is the time for the Incorporated Association of Hospital Officers to justify its existence by taking such steps as shall safeguard the interests of its members—men who have taken up the hospital service as a life work—from the intrusions of untrained and unqualified outsiders.—Yours faithfully,

Hospital Officer.

